# ‘Man Up’: the importance and strategy for placing male reproductive health centre stage in the political and research agenda

**DOI:** 10.1093/humrep/dey020

**Published:** 2018-02-08

**Authors:** Christopher L R Barratt, Christopher J De Jonge, Richard M Sharpe

**Affiliations:** 1 Reproductive and Developmental Biology, Medical School, Ninewells Hospital, University of Dundee, Dundee DD1 9SY, UK; 2 University of Minnesota, Minneapolis, MN, USA; 3 MRC Centre for Reproductive Health, The Queen’s Medical Research Institute, University of Edinburgh, Edinburgh, UK

**Keywords:** andrology, contraception, IVF ICSI, male infertility, sperm count, spermatogenesis

## Abstract

Approximately 1 in 20 young men today have sperm counts low enough to impair fertility, whereas this may not have been the case historically. The cause(s) of such a decline in male reproductive health is unknown, despite it being a global health issue. Concomitantly, little progress has been made in answering fundamental questions in andrology or in developing new diagnostic tools or alternative management strategies to ICSI in infertile men. We advocate formulation of a detailed roadmap for male reproductive health to facilitate development of a research agenda that highlights the present unmet needs and key unanswered questions, and seeks to deliver effective funding and investment to address them. This vision we term ‘a Male Reproductive Health Ecosystem’*.*

## Introduction

Whilst formulating new World Health Organization (WHO) guidelines for the diagnosis of male infertility a striking observation was the paucity of high quality data on which to base recommendations (Barratt *et al.*, 2017). Even simple questions did not have sufficient data to formulate ‘low’ let alone ‘strong’ recommendations. Similar deficiencies are evident regarding other longstanding fundamental questions in andrology. For example, spermatogenesis is absolutely dependent on high levels of testosterone, yet the mechanistic pathways via which testosterone achieves this remains a black box. This is despite our achievements in successfully manipulating androgen action specifically in every cell type in the testis via transgenesis (O’Hara and Smith, 2015). This example is symptomatic of much broader ‘andrological ignorance’, illustrated by the almost complete lack of effective treatments for male infertility or for male reversible contraception other than the condom—a stark contrast with the female, for whom there are multiple effective treatments for both infertility and contraception. This has been thrown into sharp relief by recent confirmation that sperm counts have been falling steadily for >40 years yet we do not know why (Levine *et al.*, 2017). Therefore, across the andrology spectrum, from basics to the clinic, there is a fundamental lack of knowledge that obstructs research, diagnosis and patient management. So this is where we are now, but how did we get here and how can we move forward in a structured way to improve our understanding and management of male reproductive health issues?

## The illusion of progress

Male infertility is part of a dynamic and rapidly growing health industry. ART is a worldwide, highly innovative, billion dollar enterprise. Combined with the fact that reproductive medicine is newsworthy and rapidly captures the attention of the general public, the perception from the outside is that all is well in the world of male reproduction. This is an illusion. Numerous basic clinical and scientific questions in andrology remain unanswered—some for over 50 years. A sentinel example is the lack of any real progress in developing robust methods for semen analysis, despite it being the cornerstone of infertility investigations. If a simple diagnosis cannot be correctly identified then how can we progress? These limitations have been well rehearsed elsewhere but do not diminish our collective universal failure in this area (Carrell and De Jonge, 2016). Moreover, diagnostic tools/treatments are introduced too soon, usually without proof of efficacy. A recent assessment demonstrated that the overwhelming majority of ‘add ons’ in ART (including andrology examples) had no robust evidence to support their use is damning in this respect (Spencer *et al.*, 2016). To a large extent, this situation is simply a consequence of our continuing ignorance about male infertility, as it creates a vacuum that encourages the premature introduction of new putative diagnostics assays and/or treatments because there is nothing else to offer patients. Couples seeking ART because the male partner has poor semen quality are likely to grasp at any new initiative, irrespective of cost, and are in no position to judge the benefits or efficacy. The paucity of effective non-ART treatments for male infertility, at least since 1992, simply adds to the pressure.

## Explanations for ‘andrological ignorance’—the ICSI paradox

Contrary to the negative perspective above, advances in male reproductive health have been delivered historically. We (the community of reproductive biology/medicine specialists) have a good history of basic research leading to clinical benefit, thus demonstrating that genuine advances in male reproductive health can be delivered. One example is the development of robust protocols for the cryopreservation of human sperm (Bunge and Sherman, 1953), which has had a profound impact on management of subfertile couples. However, the most transformative example was the truly remarkable development of ICSI for management of male infertility. Following the first birth of an ICSI-conceived child in 1992, ICSI use has mushroomed worldwide and is increasingly used even when no male problem exists (EIM, 2016; Boulet *et al.*, 2015). However, here’s the paradox. ICSI is not a treatment, as the man’s fertility status remains unchanged (the gametes are manipulated); it is a treatment of the female partner (encompassing ovarian stimulation, egg recovery and embryo transfer). Thus, the woman carries the treatment burden for male infertility, a fairly unique scenario in medical practice. Ironically, ICSI’s success has effectively diverted attention from identifying what causes male infertility and focussed research onto the female, to optimize the provision of eggs and a receptive endometrium, on which ICSI’s success depends. Thus, since its introduction 25 years ago, ICSI has effectively served to roadblock further scientific advancement in andrology—a widely argued viewpoint (Skakkebaek, 2017).

The situation described above has arisen over a long time period. Simply put, we are where we are so why change now?

## Five timely reasons for immediate action

The first evidence on the legacy of ICSI has emerged—sperm counts in young men conceived by ICSI are, as a group, ~50% of those in young men conceived naturally (Belva *et al.*, 2016), and as sperm counts may be a barometer of overall health and longevity (Glazer *et al.*, 2017; Hanson *et al.*, 2018), this could be a double whammy for these men. Are we being complacent about passing on other health problems for the next generation to deal with?

Significant evidence suggests that the health of future generations may be influenced epigenetically by the quality of their father’s sperm, which may have been altered by his diet and/or lifestyle (Lane *et al.*, 2014; Siklenka *et al.*, 2015); maybe such effects are the explanation for the fall in sperm counts (Levine *et al.*, 2017).

Fertile mouse sperm can now be generated *in vitro* (Saitou and Miyauchi, 2016) and the technology (Clustered Regularly Interspaced Short Palindromic Repeats-CRISPR-associated protein-9 nuclease: CrispR-Cas9) for editing DNA in gametes has arrived (Vassena *et al.*, 2016). The pressures to use both approaches in human male infertility are so substantial, that it will undoubtedly happen. As with ICSI, are we going to sit back complacently whilst it happens? Use of these techniques may have no downside for future generations, but we owe it to our children and future generations to base this on evidence rather than blind presumption, as done largely with ICSI.

In many countries, couples are delaying their first attempt at conception until the female partner is in her 30s when her fertility is declining progressively (ACOG, 2014). For example in UK, the average age at first pregnancy in 2016 was 28.8 years and 54% of all pregnancies were to mothers aged > 30 years (Office for National Statistics, 2016). Therefore, sperm quality in the male partner should be optimal to maximize chances of a pregnancy, yet sperm counts are falling (Levine *et al.* 2017) such that >15% of young men today have suboptimal semen quality, with Denmark as an example of the worst case (Skakkebaek *et al.*, 2016). Evidence of impact is that births in Denmark due to assisted reproduction (>6%; EIM, 2016) are some of the highest in the world.

**Figure 1:**
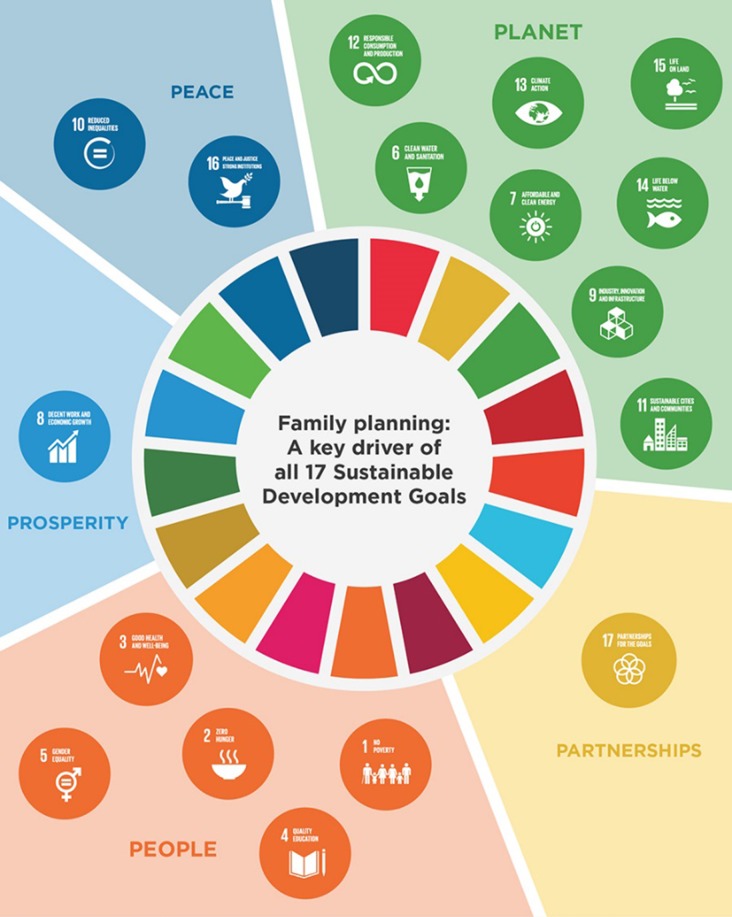
Family planning as key to success of the 17 Sustainable Development Goals. Family planning is centre stage to achieving the United Nations Sustainable Development Goals. Detailed information is provided in Starbird *et al.* (2016). See also FP 2020. Momentum at the Midpoint. 2015–2016 Progress Report. www.familyplanning2020.org. Diagram adapted from FP 2020 Progress Report.

However, the most concerning issue of all is the burgeoning world population. Population growth creates significant pressure on limited world resources and productivity and, without dramatic policy change(s), is becoming increasingly unsustainable (Gerland *et al.*, 2014; Tilman *et al.*, 2017). The United Nations (UN) Population Division’s 95% centile prediction of ~13.2 billion people by 2100 is frankly staggering and frightening in its potential impact (UN, 2017). It is remarkable then that contraceptive choices are still very limited and that the current global contraceptive strategy is suboptimal as evidenced by the continual high rates of elective terminations (Department of Health, 2016). Moreover, current strategies focus almost entirely on women. For example Family Planning 2020 (www.familyplanning2020.org), a global partnership whose aims are to increase contraception use by 120 million users by 2020, only includes women as users! No effective, reversible and widely available form of contraception has been developed for the male since the condom. Thus the burden once again falls to the female and is a price unfairly paid because of an inadequate understanding of the male reproductive process. Effective voluntary family planning is fundamental to delivery on the UN 17 Sustainable Development Goals which will transform our world (Starbird *et al.*, 2016; FP 2020; Fig. 1). To achieve this, it is critical that the reproductive input of males be considered of equal importance as females (Hardee *et al.*, 2016).

**Figure 2:**
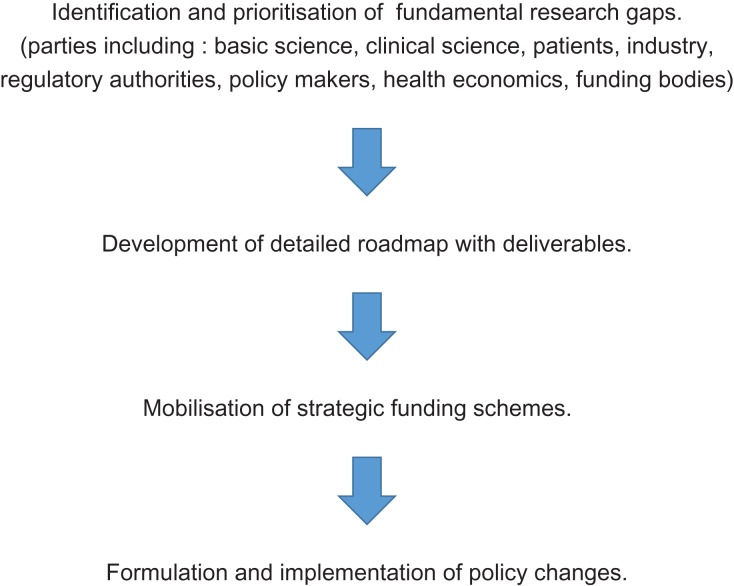
Male reproductive health ecosystem. The gaps in research are basic, translational and clinical. The proposal is to include key representatives from a spectrum of disciplines early on, for example policy experts, as in the final analysis some changes in policy strategy will be required. As for the World Health Organization (WHO) (Barratt *et al.*, 2017) this could be actioned by Expert Synthesis Groups led by a key expert. As we propose a strategic plan, overall we must be cognisant of initiatives in other areas such as growth of big data (Frégnac, 2017; Insel, 2017), whole cell maps (Horwitz and Johnson, 2017) and their biology (Kosik *et al.*, 2016). Funding for this initial approach would be required and may come from, for example, The Wellcome Trust. It is initially difficult to imagine identifying the gaps outwith the auspices of national professional societies. The default and easy route would be to get societies to do this. However, often these are talking shops and action can get stifled by political wrangling. Moreover, some work at glacial speed. An international consortium will require an international, co-ordinated effort across the discipline if it is to be effective. The proposal would work, throughout, by informing and interacting with key societies such as ESHRE, The American Society for Reproductive Medicine, the Society for the Study of Reproduction, the International Society of Andrology, and international bodies such as WHO, but not be dominated by them. Any effective strategy needs to be cognisant with what is working so far. For example, there is a renewed interest in funding work on male contraception (Bill and Melinda Gates Foundation, National Institutes of Health). Several countries have very effective networking for research delivery in infertility, e.g. The Netherlands. How did they achieve these? What can other societies/disciplines/models teach us? For example, the role of specific charities in collaboration with national funding agencies. Parkinson’s.org.uk is a good example of a charity who have a £20 million UK research commitment (Parkinson’s.org.uk) for a health issue that is less prevalent than male infertility. Effectiveness of national programmes of research should be investigated and benchmarked. Whilst any solutions involve significant new funding for male reproductive research (only ~3.6% of the UK Medical Research Council Populations and Systems panel budget was provided for male infertility research from 2014 to 2017), a piecemeal approach is not the answer (Spradling, 2016; Bloom *et al.*, 2017). The roadmap is presented in a simplistic linear fashion but there would be much dynamic movement between stages.

As outlined above, the present sidelining of andrology has arisen over many decades due to a combination of reasons. So how do we redeem the situation? We suggest the development and execution of a detailed roadmap—‘Male Reproductive Health Ecosystem’ (Fig. 2). We propose that it should encompass the areas outlined below.

## Identify research gaps

A fundamental part of a strategic roadmap is the identification of research gaps. The WHO has recently published a summary of the new evidence-based infertility guidelines (Barratt *et al.*, 2017), which could act as a foundation for developing strategies to improve research, as well as the diagnosis and treatment of male reproductive health disorders, in particular infertility. Nevertheless, it needs updating and expanding. Moreover, several critical aspects are missing from the WHO analysis. Primary among these is a detailed assessment of the economic and societal burden of male reproductive health. Many other diseases have compelling evidence of economic effects, for example infectious diseases (Bloom *et al.*, 2017), but this is all but absent for male reproductive health. A recent study (Hauser *et al.*, 2015) attempted to address this, although its focus was to suggest that this burden was attributable to endocrine disrupting chemicals, for which the evidence is rather limited. But the important point the article makes is that the cost to the European Union (EU) of male reproductive health disorders is substantial ~15 billion Euros per year. Such figures will likely surprise politicians and placing this information more into the public spotlight is one way to raise awareness and the political profile for andrology. A fundamental priority is therefore to obtain a detailed assessment of the economic and societal burden of male reproductive health disorders. This also needs to take account of the impact of infertility at a time when family size across most of the EU is below replacement level (UN, 2015), as this has huge future implications for national economics. The increasing contribution of the male to couple infertility, for reasons outlined above, provides another opportunity for moving andrology back onto the research priority stage.

## Effecting change

Once the gaps in knowledge are identified and articulated, a coherent plan for closing them is required. However, identifying the gaps in knowledge and formulating a plan are only the first steps for an effective long-term strategy. The key is for this to trigger changes in funding and, where necessary, policy. This is where it gets difficult. Evidence alone does not determine action and we need to marshal a whole series of skills to influence policy (Cairney, 2012, 2017). As Chris Tyler, who studied how evidence is used in the UK parliament, elegantly concluded ‘for research to truly inform policy, it is not enough to hope that the stars will align. The stars need to be wrestled into position’ (Tyler, 2017). One approach would be ‘a New Male Reproductive Health Ecosystem’ akin to that proposed for cancer research (Horning, 2017). This can and should involve co-ordination of a host of different parties including health care providers, patients and regulatory industries working as one interconnected community. Concomitantly, our aspirations for research financial support need to be raised logarithmically. For example, male reproductive health should be incorporated into the Global Fund to fight disease, which has been successful worldwide (Sachs and Schmidt-Traub, 2017), and aligned with worldwide challenges facing non-reproductive health of the male. Sperm counts may be a barometer for overall male health (Glazer *et al.*, 2017; Hanson *et al.*, 2018), so evidence that sperm counts have fallen 59% in ~40 years (Levine *et al.*, 2017) is a wake-up call. Baker and Shand (2017) in ‘Men’s Health: Time for a new approach to policy and practice’ show how men are ignored or given minimal priority in policy and practice to effect the UN’s Sustainable Development Goal 3 on health and wellbeing (GDG 3). Current policies are not equally applied to men and women, in part because men are reluctant to talk about their health problems, whether reproductive or not (Courtenay, 2000; Himmelstein and Sanchez, 2016).

Part of the execution strategy must involve raising the visibility of male reproductive health and engaging public support to press for change. Women could play a key partnership role in this. They have suffered invasive treatment, on behalf of male infertility management, with silent dignity in pursuit of a baby, but in a world in which we claim to be addressing inequalities between men and women, this is a stand-out example of the infringement of basic human rights and dignity. Maybe women undergoing treatment during ICSI can begin to apply pressure at the point of delivery of (their) treatment, asking ‘why can’t you treat him rather than me?’ Initiatives such as March for Science (www.marchforscience.com) are a starting platform to explain the value of male reproductive health research, building on what has been learnt from initiatives such as Andrology Australia (www.andrologyaustralia.org) who have taken the lead in ‘normalizing’ men’s health disorders, such that they can be discussed without stigmas (Hammarberg *et al.*, 2017). Urgent action is needed and under one banner—‘Man up’ (with its deliberate euphemism) is an example. There needs to be a concerted campaign to educate the public about the enormous gulf in understanding and practice regarding male versus female reproductive health, making clear this is not because all the problems are on the female side or because the female carries the biggest ‘reproductive burden’.

Undoubtedly the development and delivery of a new male reproductive health ecosystem is a daunting task. However, we need to take strength from key advantages to develop a comprehensive strategy. For example, the combination of a dynamic health industry and large public interest provides an excellent starting point from which to go forward. Moreover, in some countries, a highly innovative regulatory framework is aligned with a strong translational arm (ART industry), which should facilitate and oversee the effective execution of novel research in humans (www.hfea.gov.uk; National Centres for Translational Research in Reproduction and Infertility www.nichd.nih.gov/research/supported/NCTRI; Reproductive Medicine Network http://c2s2.yale.edu/rmn/).

## Future perspectives

Medicine currently faces the exciting challenge of incorporating new technology (e.g. stem cell therapy, gene editing), with its huge potential benefits, into patient care. To do this effectively, with minimal side-effects, understanding in a given area needs to be sufficiently advanced to enable technological advances to ‘slot in’. We are far from this position with male reproductive health, yet the new technology is almost ready to use. Unless we ‘Man up’ our research now, the present gulf in knowledge and effective therapy for infertility between male and female will grow, and it will become easier to use *in vitro* generated male germ cells than naturally produced sperm to achieve couple fertility, making infertile men truly redundant. More worrying, however, is that such applications will be founded as much on guesswork as on understanding. The future wellbeing of children resulting from assisted reproduction should not be left to chance, and we should have a clear understanding of the risks versus benefits before embracing these new developments.
